# Decreased proteasomal cleavage at nitrotyrosine sites in proteins and peptides

**DOI:** 10.1016/j.redox.2021.102106

**Published:** 2021-08-18

**Authors:** Christiane Ott, Florencia Tomasina, Nicolás Campolo, Silvina Bartesaghi, Mauricio Mastrogiovanni, Alejandro Leyva, Carlos Batthyány, Walter Meinl, Tilman Grune, Rafael Radi

**Affiliations:** a Department of Molecular Toxicology, German Institute of Human Nutrition Potsdam-Rehbruecke (DIfE), Nuthetal, Germany; b University of Potsdam, Institute of Nutritional Science, Nuthetal, Germany; c Departamento de Bioquímica, Facultad de Medicina, Universidad de la República, Montevideo, Uruguay; d Centro de Investigaciones Biomédicas (CEINBIO), Universidad de la República, Montevideo, Uruguay; e Analytical Biochemistry and Proteomics Unit, Instituto de Investigaciones Biológicas Clemente Estable & Institut Pasteur de Montevideo, Montevideo, Uruguay; f German Center for Diabetes Research (DZD), München-Neuherberg, Germany; g German Center for Cardiovascular Research (DZHK), Berlin, Germany; h Department of Physiological Chemistry, Faculty of Chemistry, University of Vienna, Vienna, Austria

**Keywords:** Proteasome activity, Protein oxidation, Nitrotyrosine, Peroxynitrite, Cytochrome *c*

## Abstract

Removal of moderately oxidized proteins is mainly carried out by the proteasome, while highly modified proteins are no longer degradable. However, in the case of proteins modified by nitration of tyrosine residues to 3-nitrotyrosine (NO_2_Y), the role of the proteasome remains to be established. For this purpose, degradation assays and mass spectrometry analyses were performed using isolated proteasome and purified fractions of native cytochrome *c* (Cyt *c*) and tyrosine nitrated proteoforms (NO_2_Y74-Cyt c and NO_2_Y97-Cyt c). While Cyt *c* treated under mild conditions with hydrogen peroxide was preferentially degraded by the proteasome, NO_2_Y74- and NO_2_Y97-Cyt c species did not show an increased degradation rate with respect to native Cyt *c*. Peptide mapping analysis confirmed a decreased chymotrypsin-like cleavage at C-terminal of NO_2_Y sites within the protein, with respect to unmodified Y residues. Additionally, studies with the proteasome substrate suc-LLVY-AMC (Y-AMC) and its NO_2_Y-containing analog, suc-LLVNO_2_Y-AMC (NO_2_Y-AMC) were performed, both using isolated 20S-proteasome and astrocytoma cell lysates as the proteasomal source. Comparisons of both substrates showed a significantly decreased proteasome activity towards NO_2_Y-AMC. Moreover, NO_2_Y-AMC, but not Y-AMC degradation rates, were largely diminished by increasing the reaction pH, suggesting an inhibitory influence of the additional negative charge contained in NO_2_Y-AMC secondary to nitration. The mechanism of slowing of proteasome activity in NO_2_Y-contaning peptides was further substantiated in studies using the phenylalanine and nitro-phenylalanine peptide analog substrates. Finally, degradation rates of Y-AMC and NO_2_Y-AMC with proteinase K were the same, demonstrating the selective inability of the proteasome to readily cleave at nitrotyrosine sites. Altogether, data indicate that the proteasome has a decreased capability to cleave at C-terminal of NO_2_Y residues in proteins with respect to the unmodified residues, making this a possible factor that decreases the turnover of oxidized proteins, if they are not unfolded, and facilitating the accumulation of nitrated proteins.

## Abbreviations

ChT-L: chymotrypsin-like
PGPH-L: peptidyl-glutamyl-peptide-hydrolyzing-like
T-L: trypsin-like
AMC: 7-Amino-4 methylcoumarin
Cyt *c*: cytochrome *c*
NO_2_Y: 3-nitrotyrosine
NO_2_Y74-Cyt c, NO_2_Y97-Cyt c: Y74- and Y97 nitrated cytochrome *c* species
UPS: The ubiquitin-proteasome system
MRM: Multiple reaction monitoring

## 1 Introduction

The ubiquitin-proteasome system (UPS) belongs to the main intracellular quality control systems, ensuring steady state levels of regulatory proteins and regulating protein turnover of dysfunctional, no longer repairable proteins. One part of the UPS is the proteasome (reviewed in Ref. [1]). The proteasome degrades unfolded, hydrophobic and mildly oxidized proteins by three catalytic activities: a peptidyl-glutamyl-peptide-hydrolyzing-like (PGPH-L), a trypsin-like (T-L) and a chymotrypsin-like (ChT-L) activity. The ChT-L activity preferentially cleaves peptide chains at the carboxyl side of the aromatic amino acids tyrosine, phenylalanine and tryptophan [1]. Sustained oxidative stress conditions [2] result in oxidatively damaged proteins that can form cross-links with other proteins and also protein aggregates which are no longer degradable by the proteasome [3]. Within these aggregates that increase during aging, also high levels of nitrated proteins (*i.e.* nitration of tyrosine to 3-nitrotyrosine, NO_2_Y) have been verified [[4], [5], [6]]. Additionally, high levels of nitrated proteins, have been found in several chronic diseases, such as Alzheimer's disease [[7], [8], [9]], multiple sclerosis [10,11], Parkinson's disease [12] and atherosclerosis [[13], [14], [15], [16]]. In addition to an increased formation, an inability in removal of nitrated proteins by the proteasome could also explain their accumulation. In biological systems, protein nitration mainly occurs by nitric oxide-derived oxidants such as peroxynitrite [17]. Peroxynitrite is formed by the diffusion-controlled reaction of nitric oxide with superoxide [[17], [18], [19]] and promotes amino acid oxidation by a variety of mechanisms. For instance, direct reactions with cysteines and methionines lead to cysteine sulfenic acid and methionine sulfoxide, respectively; alternatively, peroxynitrite-derived radicals cause the oxidation and/or nitration of amino acids such as tyrosine, tryptophan and histidine [[20], [21], [22], [23]]. In the case of tyrosine, formation of 3-nitrotyrosine and 3,3′-dityrosine are the main modifications mediated by peroxynitrite. It was previously shown that moderate concentrations of hydrogen peroxide, peroxynitrite and the peroxynitrite donor SIN-1 increased the levels of proteolytic digestion by the proteasome [24,25]; these treatments result in a variety of oxidative modifications in amino acids that, in turn, promote proteasome-dependent degradation. In contrast, the exclusive presence of 3-nitrotyrosine in peptides significantly decreased the proteolytic susceptibility to chymotrypsin [25], an observation that could be also potentially applicable in the case of the proteasome ChT-L activity, also known to cleave at tyrosine sites. In this sense, enhanced acute or chronic formation of peroxynitrite is paralleled with an accumulation of nitrated proteins [4,26,27], suggesting that their degradation may be a rather slow process. Then, the impact of protein or peptide tyrosine nitration on proteasome activity is still not established, since pure and homogenous NO_2_Y-containing substrates have not been analyzed so far. Nitration of tyrosine residues modifies side chain charge, increases amino acid volume and affects local hydrophobicity [17]. In particular, the incorporation of a nitro-group in the side chain results in a drop of the p*Ka* of the phenolic hydroxyl group from about 10 to 6.8–7.2, leading to its ionization and therefore an additional negative charge at physiologically-relevant pH [[28], [29], [30]]. The influence of these physico-chemical changes in tyrosine on the capability of the proteasome to handle tyrosine-containing peptides is far from obvious. To specifically investigate the impact of tyrosine nitration on proteasome function, degradation assays and mass spectrometry/peptide mapping analysis of purified horse heart cytochrome *c* (Cyt *c*) proteoforms in the presence of the isolated proteasome were carried out. Interactions between Cyt *c* and the proteasome have already been reported, supporting that Cyt *c* can be a useful probe for our investigations [31]. Since horse Cyt *c* is a small protein, containing only four tyrosine (Y) residues, modification by tyrosine nitration and peptide sequence analyses after proteasome digestion is much easier to perform. In this sense, we have previously characterized the peroxynitrite-dependent formation of tyrosine nitrated Cyt *c* species (NO_2_Y-Cyt c) and developed protocols for the separation and purification of site-specific tyrosine nitrated Cyt *c* proteoforms [30,32]. Interestingly, we and others have shown that nitro-oxidative stress to cells leads NO_2_Y-Cyt c formation and translocation from the mitochondria into the cytosol and nucleus [[33], [34], [35]]. Thus, with the combined use of Cyt *c* and tyrosine-containing peptides as proteasome substrates, we aimed to investigate at the biochemical level if the presence of 3-nitrotyrosine sites upsets proteasome degradation rates.

## 2 Results

### 2.1 Impact of tyrosine nitration in Cyt *c* on proteasome-mediated degradation

**Fig. 1:**
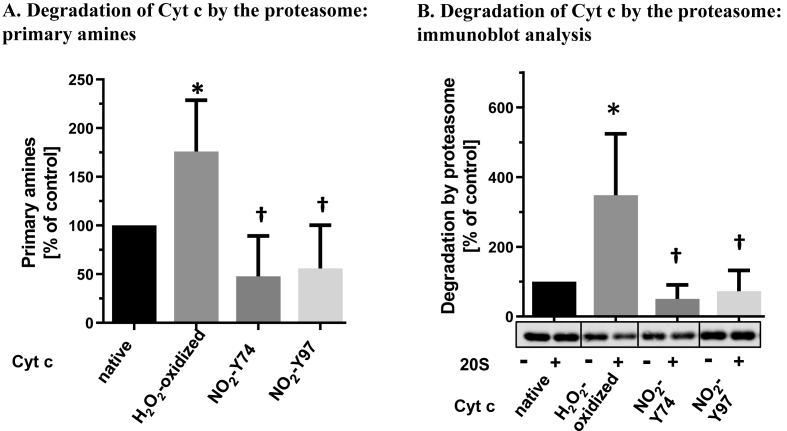
Degradation of native, H_2_O_2_-modified and nitrated Cyt *c* species by the isolated 20 S proteasome. (A) Content of primary amines 2 h after degradation of native, oxidized (100 μM H_2_O_2_) or nitrated Cyt *c* (NO_2_Y74-Cyt c and NO_2_Y97-Cyt c) by the 20S proteasome (fluorescamine assay). (B) Immunoblot analyses of oxidized (100 μM H_2_O_2_) or nitrated Cyt *c* species with (+) and without (−) the isolated 20S proteasome. Data are presented as the difference between sample with and without proteasome, as mean ± SD (at least three independent experiments). Statistical significant differences are indicated by * p < 0.05 vs. control, †p < 0.05 vs. 100 μM H_2_O_2_ using One-way ANOVA (multiple Tukey's test).

**Fig. 2:**
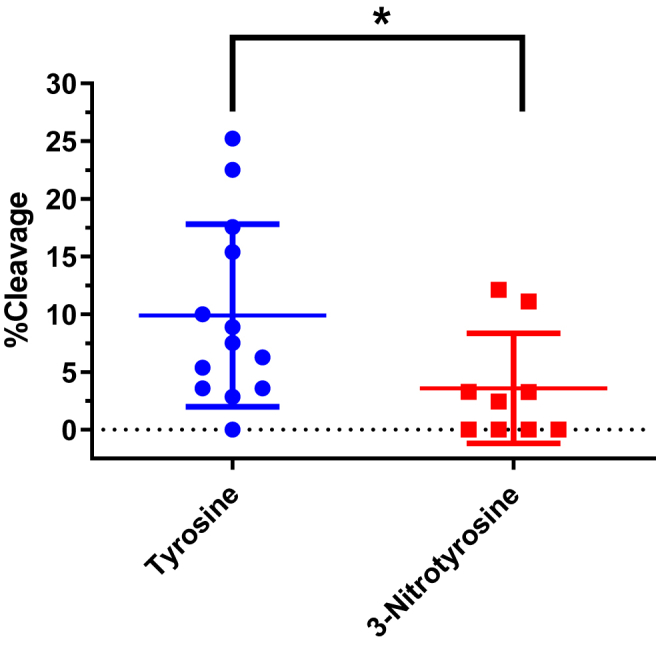
Estimation of the 20S proteasome-capabilities to cleave C-terminal to tyrosine or 3-nitrotyrosine residues in native and nitrated Cyt *c* samples by MS. The peptides resulting from the incubation of native and nitrated cytochrome *c* samples with the 20S proteasome were analyzed by nanoLC-MS/MS. The list of identified peptides was analyzed in order to calculate the % of both tyrosine and 3-nitrotyrosine proteasome cleavages (peptide bonds C-terminal to these residues) as detailed in Material and Methods. The values obtained from the analysis of the different sample types were pooled (%Y cleavage obtained from the native and two nitrated Cyt *c* species; %NO_2_Y cleavage only from the two nitrated Cyt *c* samples) and compared. Significant differences are indicated with * (p ˂ 0.05), using two-tailed Student's *t*-test.

**Fig. 3:**
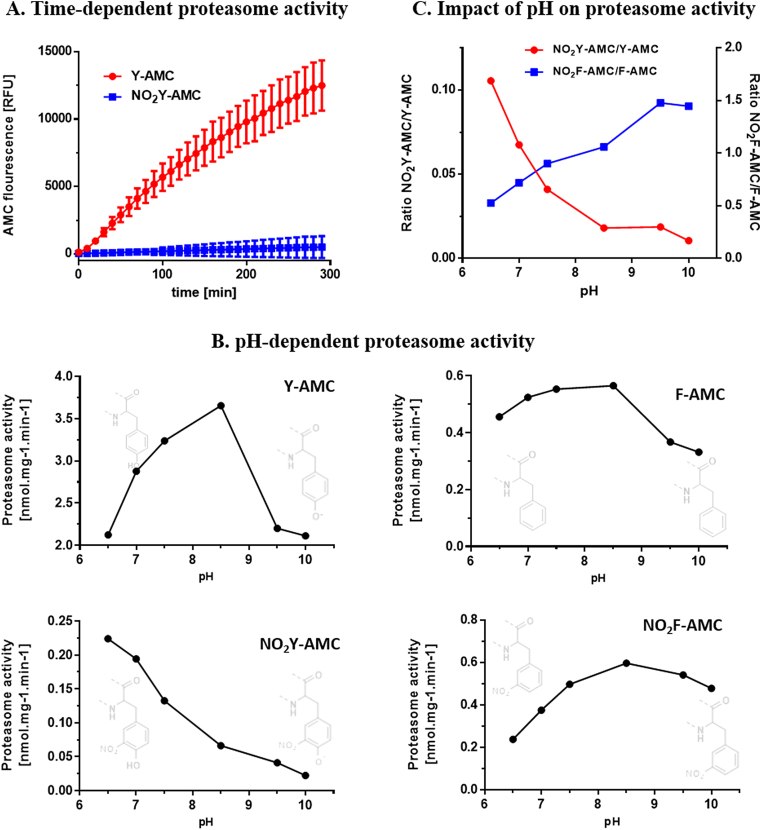
Time- and pH-dependent ChT-L 20S proteasome activity using the peptide substrates. (A) Data show the 20 S proteasome activity, using 80 μM of Y-AMC and NO_2_Y-AMC over 3 h, as measured by fluorescence of the released AMC. (B) pH-dependent proteasome activity using 80 μM of different proteasome substrates: Y-AMC, F-AMC, NO_2_F-AMC and NO_2_Y-AMC with isolated proteasome. Activity assay was performed from pH 6.5 to 10. For incubation conditions, see Material and Methods. In light gray, the structures of the main species present at each pH side are shown. (C) The plot represents the ratio of NO_2_F-AMC/F-AMC and NO_2_Y-AMC/Y-AMC ChT-L proteasome cleavage rates, performed from pH 6.5 to 10.

In order to perform initial degradation studies with the isolated proteasome, native, H_2_O_2_-oxidized and two main (mono)-nitrated Cyt *c* proteoforms were used. These two nitrated Cyt *c* (NO_2_-Cyt c) species were prepared, isolated and purified according to previous protocols of our group [30] (SI Appendix, Fig. 1A and B) and consist in a fraction composed mainly by Cyt *c* in which Y74 is nitrated (NO_2_Y74-Cyt c) and a second fraction composed by a Cyt *c* species in which Y97 is nitrated (NO_2_Y97-Cyt c). Immunochemical characterization of the two purified fractions revealed the presence of NO_2_Y (SI Appendix, Fig. 1C) in the two Cyt *c* proteoforms. A deeper characterization performed by MRM-mass spectrometry analysis after trypsin digestion revealed that, indeed, NO_2_Y74-Cyt c is mainly nitrated at Y74 (*c.a.* 50% nitration yield) with small nitration yields at Y97 and Y48, and that NO_2_Y97-Cyt c is almost exclusively nitrated at Y97 (*c.a.* 90% nitration yield), being nitration at Y48 and Y74 minimal (SI Appendix, Fig. 2, Fig. 3). In addition to tyrosine nitration, the only other modifications detected through MS-peptide mapping in purified samples were oxidation and nitration of tryptophan and oxidation of methionines, but with a minor frequency. Such species were used as an initial system for directly assessing the impact of tyrosine nitration in the context of a protein on its susceptibility to undergo proteasomal degradation.

To monitor protein degradation by the proteasome, the content of formed primary amines was determined 2 h after incubation of the different Cyt *c* species with the proteasome (Fig. 1*A*). H_2_O_2_-oxidized Cyt *c* was included in the experiments to serve as a positive control for proteasome-mediated degradation of mildly oxidized proteins [[36], [37], [38]]. Fig. 1*A* shows that levels of primary amines were significantly increased in the H_2_O_2_-oxidized Cyt *c*, compared to the control. In contrast, primary amines formation in NO_2_Y74-Cyt c and NO_2_Y97-Cyt c revealed that the exclusive presence of a NO_2_Y residue did not increase proteasomal degradation of these peroxynitrite-modified proteoforms with respect to native Cyt *c*. In fact, degradation of both mononitrated cyt *c* species showed a slight decrease with respect to native Cyt *c* and were largely decreased compared to H_2_O_2_-oxidized Cyt *c*. To confirm the results of the fluorescamine assay for primary amines, Cyt *c* degradation was additionally quantified by immunoblot analyses (Fig. 1*B*). The results analyzed by the difference of the quantity of each Cyt *c* species with and without proteasome interactions confirmed the increased degradation of H_2_O_2_–oxidized Cyt *c*, as well as the lack of such an effect for both NO_2_Y74-Cyt c and NO_2_Y97-Cyt c. These results suggest that, in the context of peroxynitrite-mediated protein modifications, nitration of tyrosine residues itself is not an oxidative posttranslational modification that increases the susceptibility of a protein for degradation by the 20S proteasome, if the substrate is not unfolded.

### 2.2 Mass spectrometry analysis of peptides after proteasome activity

To analyze the degradation products of the different Cyt *c* species (native Cyt *c*, NO_2_Y74 Cyt *c* and NO_2_Y97 Cyt *c*) after digestion with the proteasome, mass spectrometry analyses were performed. The peptide mixture resulting after the incubation of each of the samples with the proteasome was analyzed as indicated in Materials and Methods. A total number of five replicates were analyzed for each of the samples, and only the runs that yielded more than 50 Cyt *c* peptide-spectrum matches (PSMs) were considered for the analysis. A summary of the main indicators obtained for the runs is shown in Table SI; an example of the results obtained for an individual sample is shown in Table SII.

Based on the assigned peptide sequences obtained for the samples, the values of % Y cleavage for each of the three samples, as well as the % NO_2_Y cleavage for both nitrated Cyt *c* samples were estimated as explained in Materials and Methods. The pooled values obtained for the cleavage frequencies at Y and NO_2_Y sites for all of the samples were plotted in order to compare the different cleavage values (Fig. 2). As it can be seen from the calculated values, a significant smaller percentage of cleavage was obtained for NO_2_Y with respect to Y, suggesting that the proteasome has a decreased capability to cleave the polypeptide at C-terminal of 3-nitrotyrosine residues with respect to cleaving at C-terminal of tyrosine residues.

### 2.3 Measurement of ChT-L proteasome activity using tyrosine nitrated-peptide substrates

Based on our previous results, we postulated that the presence of a –NO_2_ group placed in the active center of the proteasome will inhibit or at least slow down the proteolytic degradation, perhaps due to a limited access of the substrate to the active center. To test whether this postulate is true, we performed activity assays for ChT-L activity, using the synthetic peptide substrates suc-LLVY-AMC (Y-AMC) and suc-LLV-NO_2_Y-AMC (NO_2_Y-AMC). First, we measured the release of AMC from Y-AMC and NO_2_Y-AMC by the proteasome as a function of time (Fig. 3*A*). A significantly decreased cleavage rate of the proteasome towards the NO_2_Y-AMC compared to the Y-AMC was revealed over 5h (more than twenty times lower). In order to better understand the mechanistic features that underlie this behavior, additional analyses were carried out as a function of pH, in the pH range of 6.5–10 (Fig. 3*B*). The pH dependency of the proteasome cleavage rates at the ChT-L site was also evaluated using the phenylalanine and nitrophenylalanine-analog peptide substrates, suc-AAF-AMC (F-AMC) and suc-LLV- NO_2_F-AMC (FNO_2_-AMC) (Fig. 3*B*). For the Y-AMC substrate, proteasome activity had a bell-shape curve as a function of pH, with a maximal value at pH 8.5 that was *ca.* two-fold higher than the values obtained in the acid and alkaline ends of the curve. A similar behavior was also observed for the F-AMC substrate, with identical optimal pH of the reaction, but at rates *ca.* eight times less than that obtained with the Y-AMC. However, the ChT-L activity of the 20S proteasome showed a distinct behavior towards the tyrosine-nitrated substrate (NO_2_Y-AMC) as a function of the pH; indeed, the increase of the pH of the reaction was characterized by a consistent and steep decline in the substrate degradation rates. Importantly, the proteasomal activity for NO_2_Y-AMC was ≥10 times smaller than for the Y-AMC at pH 6.5 (Fig. 3*B*), an effect that became more pronounced with the pH increase (*e.g* almost 100 times smaller at pH 8.5). On the contrary to the effect observed in NO_2_Y-AMC, the presence of a nitro group on the phenylalanine-containing substrate (NO_2_F-AMC) had a minimal effect on the proteasomal degradation rates (mostly seen at pH 6.5, where activity was *ca.* 50% less than in F-AMC); notably, the pH-dependency of the proteasome dependent degradation of NO_2_F-AMC was completely different respect to the obtained for the YNO_2_-AMC (Fig. 3*B*). To better illustrate the effect of pH on the proteasomal-dependent degradation of the tested substrate peptides, the activity ratios NO_2_Y-AMC/Y-AMC and NO_2_F-AMC/F-AMC were calculated for all pH values and plotted (Fig. 3*C*). As it can be seen, the curve obtained for the NO_2_Y-AMC/Y-AMC ratio as a function of pH suggests that the dissociation of the phenoxyl group of the NO_2_Y residue is responsible for the pH-dependent decrease on proteasomal activity towards the NO_2_Y-AMC substrate.

**Fig. 4:**
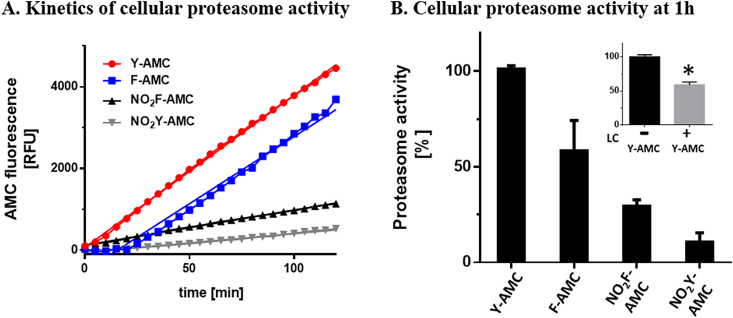
Proteasome activity-dependent cleavage of peptide substrates in lysates of U-87 MG human astrocytoma cells. (A) Data show kinetics of the cell lysates incubated with proteasome substrates Y-AMC, F-AMC, NO_2_F-AMC and NO_2_Y-AMC (all 80 μM) over 2 h. (B) Cellular proteasome activity obtained after a 1 h incubation of the four different substrates with cellular proteasome. The insert shows the degradation of Y-AMC in the absence and presence of the irreversible proteasome inhibitor lactacystin (expressed as percent of degradation in the absence of lacatcystin). Data are presented as mean ± SD, n = 3 and significant differences are indicated by *p < 0.05 vs. Y-AMC, †<0.05 vs. F-AMC and ‡p < 0.05 vs. NO_2_F-AMC using One-way-ANOVA, Tukey's comparison test.

To confirm these results in a more complex system, further activity assays were performed, using the four peptide substrates with lysates of the human astrocytoma cell line U-87 MG (Fig. 4). Kinetics clearly showed that cellular proteasome cleaves most efficiently at C-terminal of unmodified Y and F, while proteolytic activity markedly decreased by nitration (Fig. 4*A*). Using the different substrates and analyzing the slopes of released AMC-fluorescence over time showed that the reaction with Y-AMC was the fastest (37.2 RFU/min), followed by F-AMC (32.9 RFU/min), NO_2_F-AMC (8.59 RFU/min) and NO_2_Y-AMC (4.54 RFU/min). Directly comparing the cellular proteasome activity at 1 h (Fig. 4*B*) clearly shows the highest proteolytic activity with Y-AMC, followed by F-AMC. Nitration of Y and F significantly decreased the proteasome activity, compared to either Y-AMC or F-AMC. Moreover, NO_2_Y-AMC was cleaved at *ca.* 10-fold less than the unmodified peptide substrate Y-AMC and even at significantly smaller rates than NO_2_F-AMC (*ca.* two-fold difference). As a control for these experiments, cellular proteasomal activity using Y-AMC substrate was measured in the presence of the cell-permeable and irreversible proteasome inhibitor lactacystin (LC) [[39], [40], [41]], leading to a significant inhibition of proteasomal activity (Fig. 4*B insert*). This observation confirms that the measurements made with the cellular extracts correspond the proteolytic activity of the proteasome.

### 2.4 Degradation susceptibilities of the Y-AMC and NO_2_Y-AMC substrates by proteinase K

**Fig. 5:**
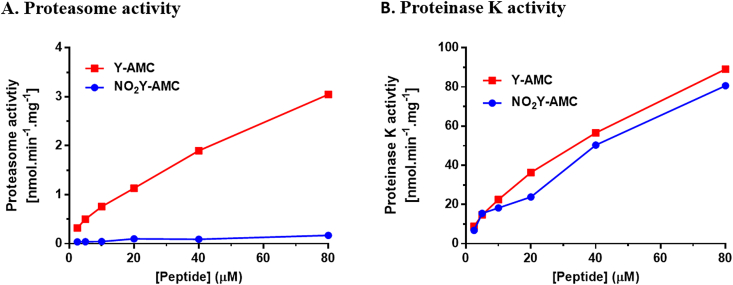
Comparison of proteasome and proteinase K cleavage rates using Y-AMC and NO_2_Y-AMC peptides. Data represent concentration-dependent proteasome and proteinase K activity using Y-AMC and NO_2_Y-AMC peptides as substrates, up to a concentration of 80 μM for 1 h. Data is shown in nmol.min-1.mg-1.

To finally validate that the decreased AMC release measured was caused by the inability of the proteasome to cleave the nitrated substrate, and not due to intrinsic molecular factors of the artificial substrate (e.g. fluorescence quenching by the –NO_2_Y moiety, modulation of the peptide bond reactivity, others), we performed activity assays using different concentrations of Y-AMC and NO_2_Y-AMC (0–80 μM) with either proteasome or proteinase K (Fig. 5). Proteinase K is a non-related fungal serine-protease that can cleave peptide bonds at C-terminal of aromatic residues [42], meaning that it will also be able to release the AMC fluorophore from the Y-AMC and NO_2_Y-AMC substrates. In this sense, if proteinase K can cleave both substrates with similar rates, it will confirm that the observed results in the previous section are actually due to a decreased capability of the proteasome to perform the cleavage at C-terminal of NO_2_Y residues. While proteinase K was able to release the same amount of AMC from the nitrated as from the unmodified peptide, the amount of AMC released from the nitrated peptide by the proteasome was distinctly smaller than from the unmodified peptide, as previously measured. These results confirm that the decreased ChT-L activity observed towards NO_2_Y-containing peptide substrates with respect to their native forms is due to a decreased ability of the proteasome to cleave at C-terminal of the NO_2_Y residue.

## 3 Discussion

It is currently accepted that mild or moderate oxidative post-translational protein modifications lead to enhanced recognition and degradation by the 20S proteasome and that this constitutes a mechanism of removal and turnover of non-native proteins [24,36,[43], [44], [45], [46]]. Indeed, amino acid oxidation usually leads to enhanced surface hydrophobicity and partial unfolding, characteristics that favor proteasome-dependent (and ATP-independent) protein degradation [[47], [48], [49], [50]]. However, analysis of the precise effect of specific amino acid modifications is scarce in the literature and, in particular, there is a paucity of information on the specific impact of tyrosine nitration to 3-nitrotyrosine as a molecular feature for proteasome-dependent degradation. The effects of tyrosine nitration are far from obvious for a variety of reasons. Nitration alters the physico-chemical properties of a tyrosine residue in different aspects: increase in residue volume and hydrophobicity and a notably decrease in the phenolic –OH pKa value from ca. 10 to 6.8 (for free tyrosine residues, it may vary for tyrosine residues in proteins). This last alteration results in the existence of nearly equal amounts of deprotonated (negatively charged) and protonated (neutral) phenolic side chains under physiological conditions, and so, the simultaneous presence of different functional/structural consequences. If the NO_2_Y residue exists in the protonated neutral form, its hydrophobicity is increased with respect to the unmodified Y residue, while if it exists in the deprotonated negatively-charged form, the opposite may occur. This means that the consequences of the nitration of a single Y residue within a protein may vary considerably, leading to different possibilities of structural alterations of the protein (e.g. movement of the modified residue to more exposed or more buried regions of the protein) [17], which may then render different susceptibilities for proteasomal degradation. Besides this inherent complexity, protein tyrosine nitration, either in vitro or in vivo, usually occurs with other oxidative modifications, making then the analysis of the specific effect of tyrosine nitration on proteasomal degradation much harder.

To assess how tyrosine nitration specifically affects the degradation susceptibility of a protein by the 20S proteasome, native, H_2_O_2_-oxidized and nitrated Cyt *c* species (NO_2_Y74-Cyt c and NO_2_Y97-Cyt c) were incubated with purified proteasome. While 100 μM H_2_O_2_ increased degradation of Cyt *c* by proteasome, the presence of a single NO_2_Y residue (NO_2_Y74-Cyt c or NO_2_Y97-Cyt c) did not enhance the proteasomal degradation of Cyt *c*, suggesting that nitration of a single tyrosine residue in Cyt *c* does not represent a molecular signature that promotes its proteasomal degradation, obviously due to the fact that the protein structure is not totally disrupted. Previous studies reported that peroxynitrite treatment of lysozyme, which dose-dependently induces nitration of its tyrosine residues, significantly increased its proteasomal degradation in vitro [51]. Despite this apparent contradictory finding, it is worth mentioning that peroxynitrite-treated lysozyme most likely contains several nitro-oxidative modifications beyond tyrosine nitration, so it is possible that its increased proteasomal degradation is a consequence of multiple-site protein modification. In order to shed light more precisely into the specific effect of tyrosine nitration on NO_2_Y74-Cyt c and NO_2_Y97-Cyt c proteolytic degradation, HPLC-MS/MS peptide-mapping analysis was performed on the product mixture to identify the different peptides that were generated (SI Appendix, Tables I and II). Through this approach, it is possible to have an overall idea about how efficiently the chymotrypsin-like activity (ChT-L) of the proteasome is able to cleave peptide bonds at C-terminal of Tyr residues and NO_2_Tyr residues of the native and nitrated substrates (as described in Materials and methods). By analyzing the different PSMs obtained for all the protein samples incubated with the 20S proteasome (Cyt *c*, NO_2_Y74-Cyt c and NO_2_Y97-Cyt c), a significant decrease in the % Cleavage was observed at C-terminal of NO_2_Tyr residues with respect to unmodified Tyr residues (Fig. 2). This finding suggests that the ChT-L activity of the proteasome has a lower capability to cleave sites at C-terminal of NO_2_Tyr residues within proteins than at C-terminal of Tyr residues. Nevertheless, this result has to be taken with care, since the values represent an estimation and there are some considerations that have to be noted. First of all, the MS analysis of proteasome-digested proteins may give a limited insight of the digestion process, since this technique does not allow to identify small peptides (from 2 to 4 amino acids). Considering that the proteasome has multiple catalytic activities and is able to cleave at many different positions, the amount of free amino acids and small peptides could be of relevance, and the information contained in such species could either enhance or soften the observed differences. Second, the approach used for estimating the cleavage percentages is quite limited and arguable, as usually happens with label-free quantification by mass spectrometry. In spite of this, several works have shown that spectral counting-based quantification represents a simple and useful approach in proteomics for relative quantification [[52], [53], [54], [55]]. Finally, it has to be considered that the experimental design used in our research aims to establish the specific role of tyrosine nitration on the proteasomal degradation of a protein; this means that the conditions under which the digestion of the protein occurs is not necessarily optimal (as would be, for instance, denaturing conditions), resulting in low yields of peptide generation. This, in conjunction with the very high number of possible peptides that can arise from the proteasomal degradation, makes that the amount of PSMs detected for the samples is a bit lower than desired. Independently of all these considerations, the result obtained herein can be considered as a first mass spectrometry-based approach indicating that, despite the overall effects on total protein degradation (Fig. 1), the proteasome has a decreased capability to cleave C-terminal of 3-nitrotyrosine with respect to C-terminal of tyrosine. It is important to note that this does not exclude the possibility that, within the protein, the nitration of a specific Tyr residue could induce structural alterations that increase the recognition of the protein for proteolytic degradation by the proteasome. The possible simultaneous occurrence of both phenomena could explain why the proteasomal degradation of both NO_2_Y74-Cyt c and NO_2_Y97-Cyt c was not significantly different from that of native Cyt *c* (Fig. 1).

To verify more clearly that proteasome ChT-L activity at C-terminal of Tyr residues is affected by nitration, activity assays were performed using the proteasome substrate Y-AMC and its specifically modified form, NO_2_Y-AMC (Fig. 3). Comparing both substrates, we obtained a large difference in enzyme activity over 3 hs (Fig. 3A), further supporting the previous result that suggested a decreased capability to cleave at C-terminal of NO_2_Tyr. This result contrasts with the reports of Curry-McCoy et al. [51], in which the 20S proteasome was seen to degrade more actively peroxynitrite-treated Y-AMC substrate than its unmodified form. Again, it is worth considering that this modified substrate is probably not equivalent to a synthetic NO_2_Y-AMC (as used in this work), as peroxynitrite exposure of the Y-AMC substrate may lead to a mixture of different modified species, which include modifications other than nitration of the tyrosine residues of the substrate. Although the possible modifications are, for this substrate, much less than for a protein, these considerations need to be taken into account in order to explain the different behavior found in our work. To figure out why the proteasome seems to be unable to handle nitrated peptides, we further analyzed whether the different pKa of 3-nitrotyrosine with respect to tyrosine might be responsible (Fig. 3B and C). If the dissociation state of the tyrosine hydroxyl group is important for proteasomal degradation, a difference in the pH dependency should be observed for the degradation of Y-AMC and NO_2_Y-AMC. In contrast, the degradation of the analogous phenylalanine- and nitrophenylalanine-containing substrates (F-AMC and NO_2_F-AMC) should be largely pH-independent due to the lack of a hydroxyl group. Since the nitro group at position 3 in the NO_2_Y-AMC drastically lowers, due to its strong negative inductive effect, the pKa of the phenolic group at position 4 (pKa NO_2_Y = 6.8–7.2 [28,29]), a considerable amount of NO_2_Y residues are negatively charged at physiological conditions, while unmodified Y-AMC (pKa Y = 10.5) remains protonated and thus uncharged. Therefore, we measured the ChT-L activity of the proteasome at varying pH-values in order to assess the effect of the phenolic –OH deprotonation in the decreased ChT-L activity towards the NO_2_Y-AMC substrate. These experiments clearly showed that the overall low activity against NO_2_Y-AMC dropped from pH 6.5 (≈pKa) with increasing pH-values, indicating that, indeed, the proteasome did not accept the negatively charged peptide as a substrate. In contrast, the cleavage of the Y-containing peptide was slightly enhanced as the pH increased, decreasing only at the most basic tested pH values (9.5 and 10). However, this was also observed for the F-AMC substrate, which lacks the phenol group, suggesting that the decrease at these most basic pH values is actually more related to an alteration of the function of the proteasome itself (due to its protein nature) and not to substrate alterations. On the other hand, the fact that the proteasomal activity was stable over the 6.5–8.5 pH range using the F-AMC substrate further suggests that, indeed, the pH-dependent changes at the recognition/cleavage sites of the substrate are responsible for the lower activities measured in that range, and not pH effects on the proteasome.

According to Riordan et al. [28] and Sokolovsky et al. [29], the pKa for the phenoxyl group of free NO_2_Y is in the range 6.8–7.2; even though this value might vary for the nitrated tyrosine in the NO_2_Y-AMC substrate, one would expect that, at pH 6.5, nearly 50% of the phenol groups are ionized (phenolate), giving a net negative charge to the residue. However, at pH 6.5, the proteasomal ChT-L activity towards the NO_2_Y-AMC substrate was decreased to approximately 10% of that measured for the unmodified Y-AMC substrate (Fig. 3C); this means that phenolic dissociation of the NO_2_Y-AMC due to a decreased pKa is not the only feature that impaired proteasomal ChT-L activity. In this sense, it is most likely that the steric effects imposed by the presence of the bulky –NO_2_ group are also involved in the decreased ChT-L activity of the proteasome towards the tyrosine-nitrated substrate. Although it would be possible to partially assess this latter effect through the comparison of the F-AMC and NO_2_F-AMC substrates, the ratio of NO_2_F-AMC/F-AMC degradation varied much less and following a different behavior over the pH range (Fig. 3C). At pH values ranging from 6.5 to 7.5, the NO_2_F-AMC was degraded at lower rates than its unmodified version, but the opposite happened at the range 8.5–10 (Fig. 3C); this suggests that the steric effects of the -NO_2_ group are not that obvious in the nitrophenylalanine motif regarding proteasomal ChT-L inhibition, although this does not necessarily represents the same as for nitrotyrosine. Further studies are needed to define at which level of catalysis is nitration of tyrosine residues affecting proteasomal degradation (i.e. substrate accessibility, binding or catalysis).

To verify whether the same effects can be revealed with cellular proteasome, we repeated the activity measurements on the peptide substrates using cell lysates from the human astrocytoma cell line U-87 MG (Fig. 4). Several proteomic approaches have shown that, as well as heart and skeletal muscle, the brain contains a high content of nitrated proteins in aging and in various neurodegenerative diseases, such as Tau protein [56], α-synuclein [57] and glyceraldehyde 3-phosphate dehydrogenase [9]. Additionally, the brain (particularly, the cerebellum) produces highest levels of nitric oxide [58], making the central nervous system (CNS) more susceptible to nitrosative stress, especially in aging [59]. To handle the elevated stress levels, proteasome is highly present in all cells of the CNS and mainly appropriate for intracellular protein degradation, particularly in the glia cells [60]. Proteasome inhibition and dysfunction alone is sufficient to induce neuronal cell death [61]. Since astrocytes are responsible for neuronal protection, the cells need a well-endowed antioxidative protection, including high proteasome levels. Performing the proteasome activity assay with U-87 MG astroglial cell lysates and Y-AMC or NO_2_Y-AMC substrates, we also obtained a significantly decreased release rate of AMC from NO_2_Y-AMC compared to Y-AMC (Fig. 4). The calculated slope clearly demonstrates the impact of the different substrates on proteasome ChT-L activity in the cells. Comparing Y-AMC with F-AMC, activity with the latter was again smaller, confirming the results with the isolated proteasome and indicating that the ChT-L activity of the proteasome is actually sensitive to subtle structural changes in the substrate (i.e. the presence of a –OH phenolic group). Modifying F-AMC by nitration led to an even stronger decline in enzyme activity, supporting in this case the previously mentioned hypothesis that the presence of a –NO_2_ group, without inducing the ionization of the residue, also impacts proteasome ChT-L activity, possibly through altering residue volume, hydrophobicity or electrostatic properties. Finally, the strongest decrease in proteolytic activity was again obtained using NO_2_Y-AMC, demonstrating that, besides changes in substrate structure and electrostatic properties, the ionization also affects the ChT-L activity.

In conclusion, our investigations show that tyrosine-nitrated peptides and proteins are poorer substrates for the ChT-L activity of the proteasome, both due to the steric and electrostatic alterations that nitration induces on the residue, as well as by favoring the ionization of the phenolic –OH of the side chain at physiological pH values. It has to be tested whether our finding can be related to the fact that nitrated proteins tend to accumulate and aggregate in cells and tissues during pathological processes and aging [4,9,15,[62], [63], [64], [65]]. Nevertheless, it is important to remark that, within a protein, tyrosine nitration can concur with other oxidative modifications and/or trigger structural changes with may promote the proteosomal recognition and activity by, for example, the exposure of hydrophobic patches. Overall, our studies underscore that 3-nitrotyrosine per se blunts proteasome-dependent protein turnover.

## 4 Materials and methods

### 4.1 Synthesis and purification of native, tyrosine nitrated and oxidized Cyt *c*

Horse heart holo-Cyt c (192–10, Lee Biosolutions) was nitrated and purified as described in Ref. [30]. Briefly, nitration of Cyt *c* was performed by reaction with peroxynitrite. Peroxynitrite was added to cytochrome *c* (1 mM) in 200 mM potassium phosphate, 25 mM sodium bicarbonate and 100 μM DTPA at pH 7.0 and 25 °C as a continuous flux (0.13 mM.min-1) using a motor-driven syringe (SAGE Instruments, Boston, MA) as described previously [66], under vigorous vortexing to circumvent mixing artifacts. The reaction mixture was passed through a cation exchange sulfopropyl-TSK preparative column (21.5 mm3, 15.0 cm; Tosoh Biosep) at a low rate of 3 ml.min-1. Column was equilibrated with a 5 mM ammonium acetate buffer (pH 9.0), kept for 5 min in this buffer and then eluted using linear gradients of the same salt from 5 mM to 150 mM (5–30 min) and from 150 mM to 400 mM (30–75 min). The resulting HPLC profile (Supporting Information, Fig. S1A) was subjected to mass spectrometry analysis and peptide mapping [30] and immunochemical analysis (Fig. S1C) and validated the presence of native and two main tyrosine (mono)nitrated Cyt *c* fractions, from here on referred to as NO_2_Y74-Cyt c and NO_2_Y97-Cyt c (Fig, S1B). The Cyt *c* proteoforms that were utilized for the proteasome degradation studies were subjected to a re-purification by a second round of cation exchange chromatography. After this last purification step, and in order to better characterize the modification degree of the two different nitrated cytochrome *c* species, multiple reaction monitoring (MRM)-mass spectrometry experiments were designed from the previously MS-identified sequences obtained after trypsin digestion of the samples. This targeted assay allowed to estimate the nitration ratio of each of the four tyrosine residues in cytochrome *c*, by simultaneously detecting both the modified (nitrated) and unmodified variants of each tyrosine-containing peptide, as a method for validating the purification of the Cyt *c* proteoforms (Figures S2 and S3). Cytochrome *c* was also oxidized by H_2_O_2_ treatment as in previous works [67,68]; specifically, 100 μM Cyt *c* was incubated with 100 μM H_2_O_2_ for 2 h at room temperature in 30 mM Hepes + 300 mM NaCl, pH 7.5. The reactions were terminated by addition of 100 U of catalase for 15 min. The 1/1 M ratio of Cyt *c* and H_2_O_2_ was conceived to cause a mild oxidation of the protein, resulting on proteoforms containing oxidized Tyr67, Met80 and Lys72/73 to 3,4- or 2,4-dihdroxyphenylalanine, methionine sulfoxide and aminoadipic semialdehyde, respectively [68].

### 4.2 Degradation of Cyt *c* by the proteasome: fluorescamine and immunochemical assays

Purified mono-nitrated and H_2_O_2_-oxidized Cyt *c* species (50 μg each) were incubated with 8 μg isolated proteasome, obtained from human red blood cells (as described in Ref. [69]), for 2 h at 37 °C in 30 mM Hepes, 300 mM NaCl, pH 7.5. Afterwards, the fluorescamine assay was performed as described by Hohn et al. [70], where the obtained primary amines react with fluorescamine (Fluram, Sigma, 47614) to form a fluorescent product, measurable at λex = 360 nm, λem = 460 nm. Proteasome degradation was determined as the difference between sample with and without proteasome to avoid distortions by proteasome-independent formation of free amines as a consequence of oxidant treatment. For immunochemical analyses, 1 μg protein extract of samples with and without proteasome was added to Laemmli buffer and separated using SDS-PAGE (BioRad, Munich, Germany). In the case of dot blots, lysates were dropped on the nitrocellulose and subsequently allowed to dry at room temperature. For immunodetections, the following antibodies were used: *anti*-Cyt c antibody (#136F3, Cell Signaling) and anti-NO_2_Y antibody (ab110282, Abcam). Fluorescent-conjugated secondary antibodies (Li-COR Biosciences) were used according to the specifications of the manufacturer and membranes were scanned with the Odyssey Infrared Imaging System (LI-COR Biosciences, Bad Homburg, Germany). Blot quantifications were calculated, using the Image Studio™ Software (LI-COR). According the instructions of the manufacturers, data for the images were captured across a dynamic range without image saturation, “blowout”, or sacrificing sensitivity.

### 4.3 Mass spectrometry analyses

Native-, NO_2_Y74- and NO_2_Y97-Cyt c species (50 μg each) were digested by 8 μg proteasome (2h, 37 °C) to obtain peptides to be analyzed by mass spectrometry (six samples per condition). After 2h, proteasome was precipitated with 10% trichloroacetic acid (TCA) and after centrifugation (5 min, 3000 g) the supernatant with the peptides was taken for solid phase extraction (SPE) to remove remaining TCA. For SPE, a C18 column (Chromabond C18, 3 ml/500 mg) was used, conditioning 1.3 ml of 0.1% formic acid (FA) in methanol and 5 ml of 0.1% FA in water. Then, 500 μL of each of the samples was added and passed through the column by vacuum. Subsequently, the column was washed with 3 ml of 0.1% FA in water and sample was eluted with 1.5 ml of 0.1% FA in methanol. Afterwards, samples were desiccated in a SpeedVac (Thermo Scientific) until they were completely dry. Peptides were separated using a nano-HPLC (EASY-nLC 1000, Thermo Scientific) coupled to an LTQ Velos mass spectrometer (Thermo Scientific). Peptide mixtures were injected into an Acclaim® PepMap C18 nano-trap column (75 μm × 2 cm, Thermo Scientific) and separated on a 50 μm × 150 mm C18 Easy spray column (PepMap® RSLC, 2 μm, 100 Å) at a flow rate of 250 nL/min. Peptide elution was achieved with a 100 min gradient from 5% to 55% of mobile phase B (A: 0.1% formic acid; B: 0.1% formic acid in acetonitrile). Online MS analysis was carried out in a data dependent mode (positive mode, full scan using a mass range of 300–2000 *m/z* followed by MS/MS of the top 10 ions in each segment) using a dynamic exclusion list (exclusion duration 45 s). Electrospray voltage was 1.40 kV and capillary temperature was 200 °C. Peptides were identified by searching in Proteome DiscovererTM using a customized database with the following parameters: no-enzyme (unspecific) cleavage, two maximum missed cleavage sites, 1.5 and 1.0 Da precursor and fragment mass tolerance, respectively. We searched for nitro-tyrosine, nitro-tryptophan, oxidized cysteine and oxidized methionine as variable modifications.

The different peptides identified for each sample were analyzed in order to estimate a percentage of the proteasomal cleavage at the C-terminal of tyrosine and 3-nitrotyrosine residues as indicated below. All of the analyzed samples identified a variable amount of cytochrome c-derived peptides; only the samples that yielded more than 50 peptide-spectrum matches (PSMs) were considered for further analysis, in order to calculate a more trustable estimate of proteasomal cleavage. The percentage (frequency) of cleavage after tyrosine residues (% Tyr cleavage) for each sample was calculated by adding all the PSMs identified for every peptide containing a C-terminal tyrosine residue (i.e. the peptide was originated by the cleavage of the proteasome after such tyrosine residue) and dividing such count by the sum of all tyrosine containing PSMs found for the sample (i.e. all tyrosine-containing peptide counts identified, both at C-terminal positions or elsewhere). This value yields an estimate, from the MS-identified peptides derived from the digestion of both native and modified cytochrome *c* by the proteasome, of the ability of the proteasome to cleave at the C-terminal of a tyrosine residue. The percentage of cleavage after 3-nitrotyrosine residues (% NO_2_Tyr cleavage) for each NO_2_Tyr-containing Cyt *c* sample was calculated analogously to the % Tyr cleavage, but considering in this case only the spectral counts of NO_2_Tyr-containing peptides (this is, the total PSMs containing a C-terminal NO_2_Tyr, divided the sum of all NO_2_Tyr-containing PSMs, either the NO_2_Tyr is at C-terminal or not). After obtaining the % Tyr cleavage (for both native and NO_2_Tyr74 and NO_2_Tyr97 Cyt *c* samples) and the % NO_2_Tyr cleavage (for the NO_2_Tyr74 and NO_2_Tyr97 Cyt *c* samples), the individual values were plotted for comparison.

### 4.4 Synthesis of NO_2_Y-AMC

The Suc-Leu-Leu-Val-3-NO_2_Tyr-AMC (NO_2_Y-AMC) peptide substrate used as a probe for studying the degradation of tyrosine-nitrated peptides was synthesized as follow. A 2-Chloro-Trityl chloride resin was loaded with Fmoc-Tyr (NO_2_)–OH (3 eq) in the presence of N,*N*-diisopropylethylamine (DIPEA, 7,5 eq) for 30 min. After two washing steps with dichloromethane (DCM) remaining reaction sides were capped using a mixture of DCM/methanol/DIPEA (80/15/5, v/v/v, 2 times, 10 min each). The remaining amino acids were coupled using the standard Fmoc/tBu-strategy and in situ DIC/HOBT activation. Since the unprotected hydroxyl group of Fmoc-(Tyr (NO_2_)–OH) could have reacted with the resin's reaction side providing free α-carboxy groups, propylamin was coupled first in order to block those. Succicate (suc) was incorporated as mono-*tert*-Butyl succinate.

The peptide was cleaved off the resin as protected compound by incubation with a mixture of acetic acid/trifluoroethal/DCM (1/1/8, v/v/v) for 30 min. The peptide was precipitated in *n*-hexane and dried in vacuum. 18 mg of the raw product were redissolved in 3 ml dimethylformamide and incubated with 7-amino-methylcoumarin, DIC and HOBT (0,36 mmol each) for 2 h. After the mixture was dried in vacuum, deprotection of the N-terminus was carried out using TFA and supplemented with a scavenger mixture (12.5% v/v; ethandithiole, m-cresol, water, and thioanisole, 1/2/2/2 v/v/v/v) followed by precipitation in ice cold diethylether. The peptide was purified by RP-HPLC on a Jupiter C18-column (ID: 10 mm) using a linear aqueous acetonitrile gradient containing TFA (0.1% v/v) as ion pair reagent. In order to verify the analyses, we repeated the measurements with standardized peptides from Bachem, Suc-Leu-Leu-Val-Tyr-AMC (4011369.0025), Suc-Leu-Leu-Val-3-nitro-Tyr-AMC (4107824) and Suc-Leu-Leu-Val-3-nitro-Phe-AMC (4110120).

### 4.5 Measurement of proteasome and proteinase K activity

U-87 cells were cultured under standard conditions as described in Ref. [71]. Cells were harvested by trypsinization and lysed in 1 mM DTT passing lysates 20 times through a 27-gauge needle attached to a 1 ml syringe and centrifugation at 12,000 g for 15 min, 4 °C to remove non-lysed cells and membrane debris. Supernatants were used for determination of protein, using Bradford assay, and proteasome activity. For the proteolytic activity measurements, peptide substrates were incubated with either 1 μg isolated proteasome or 10 μg cell lysate (cellular proteasome) or 0.1 U of proteinase K (sigma Aldrich, P6556). To perform the measurements at various pH values, different ratios of Na_2_HPO_4_ (0.5 M) and KH_2_PO_4_ (0.5 M) were used to obtain the appropriate pH. For the measurements at different concentrations we used a ratio of Na_2_HPO_4_ (0.5 M) and KH_2_PO_4_ (0.5 M) to obtain a pH of 7.0. Final pH of the reaction was controlled under all experimental conditions. The peptide substrates used for the various measurements of ChT-L activity of the proteasome and proteinase K activity were Suc-LLVY-AMC and its modified form NO_2_Y-AMC, Suc-LLVNO_2_F-AMC (all obtained from Bachem) and Suc-AAF-AMC (F-AMC, S8758, Sigma Aldrich). Different concentrations of the substrates were added to the isolated or cellular proteasome and proteinase K for 1–3 h at 37 °C. As a standard for quantification, AMC (Enzo, BML-KI107-0001) was used. AMC liberation from the substrates was measured with a fluorescence reader at λex = 360 nm, λem = 460 nm.

### 4.6 Statistical data analysis

Data of all panels represent the mean ± SD of at least three independent experiments. Statistical data analysis was performed either using Student's *t*-test (directly comparing two samples) or One-way ANOVA (multiple Tukey's test, comparing >2 samples), p < 0.05 was considered significant.

## Declaration of competing interest

None.
