# Supplementary material for: Decreased proteasomal cleavage at nitrotyrosine sites in proteins and peptides
Source: Redox Biol. 2021 Aug 18;46:102106. doi: 10.1016/j.redox.2021.102106 (PMC8403764; doi:10.1016/j.redox.2021.102106)
Supplement: Multimedia component 1 [file mmc1.pptx]

## Slide 1
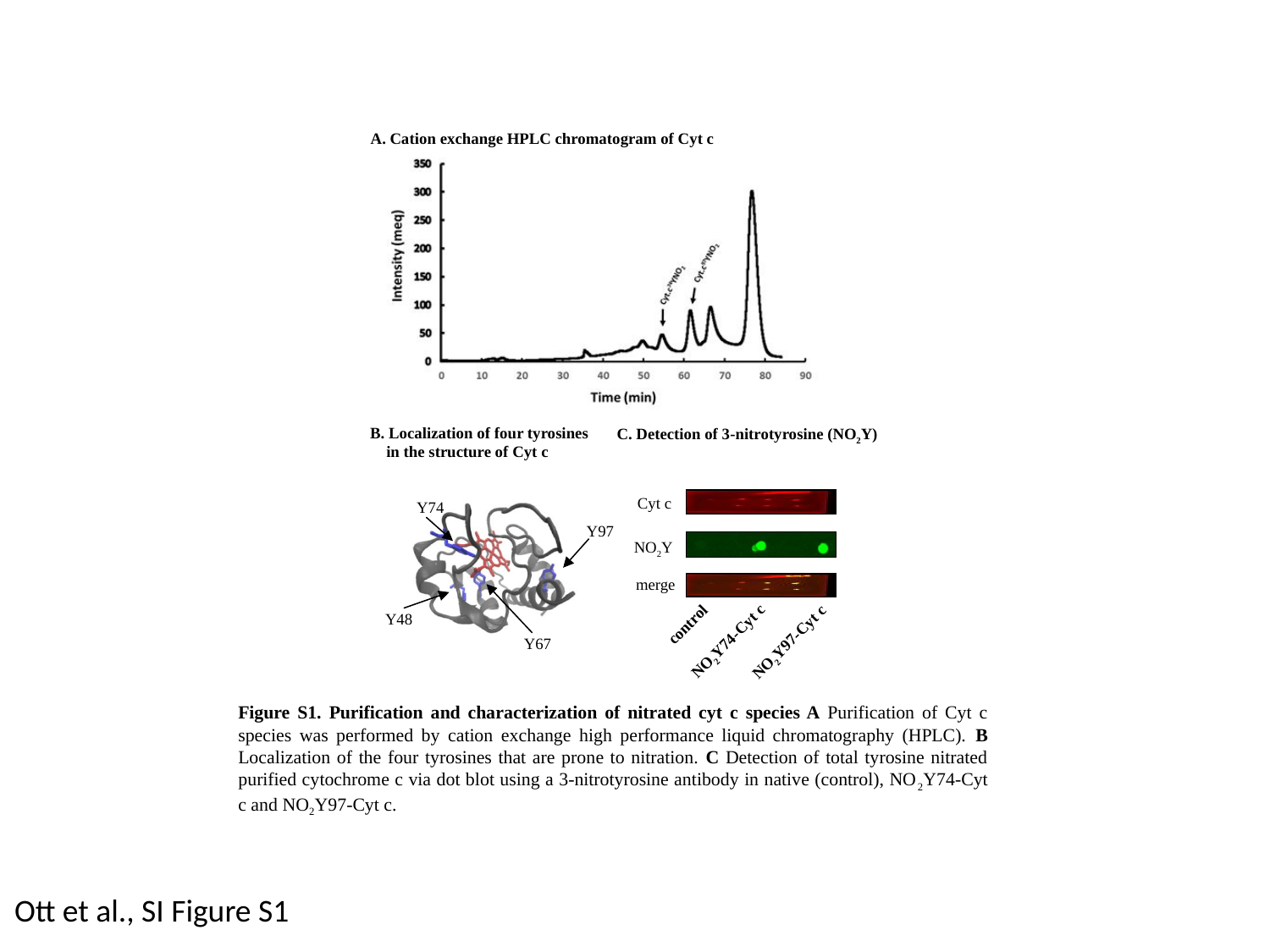

A. Cation exchange HPLC chromatogram of Cyt c
B. Localization of four tyrosines
 in the structure of Cyt c
C. Detection of 3-nitrotyrosine (NO2Y)
Cyt c
Y74
Y97
NO2Y
merge
Y48
control
NO2Y74-Cyt c
NO2Y97-Cyt c
Y67
Figure S1. Purification and characterization of nitrated cyt c species A Purification of Cyt c species was performed by cation exchange high performance liquid chromatography (HPLC). B Localization of the four tyrosines that are prone to nitration. C Detection of total tyrosine nitrated purified cytochrome c via dot blot using a 3-nitrotyrosine antibody in native (control), NO2Y74-Cyt c and NO2Y97-Cyt c.
Ott et al., SI Figure S1

## Slide 2
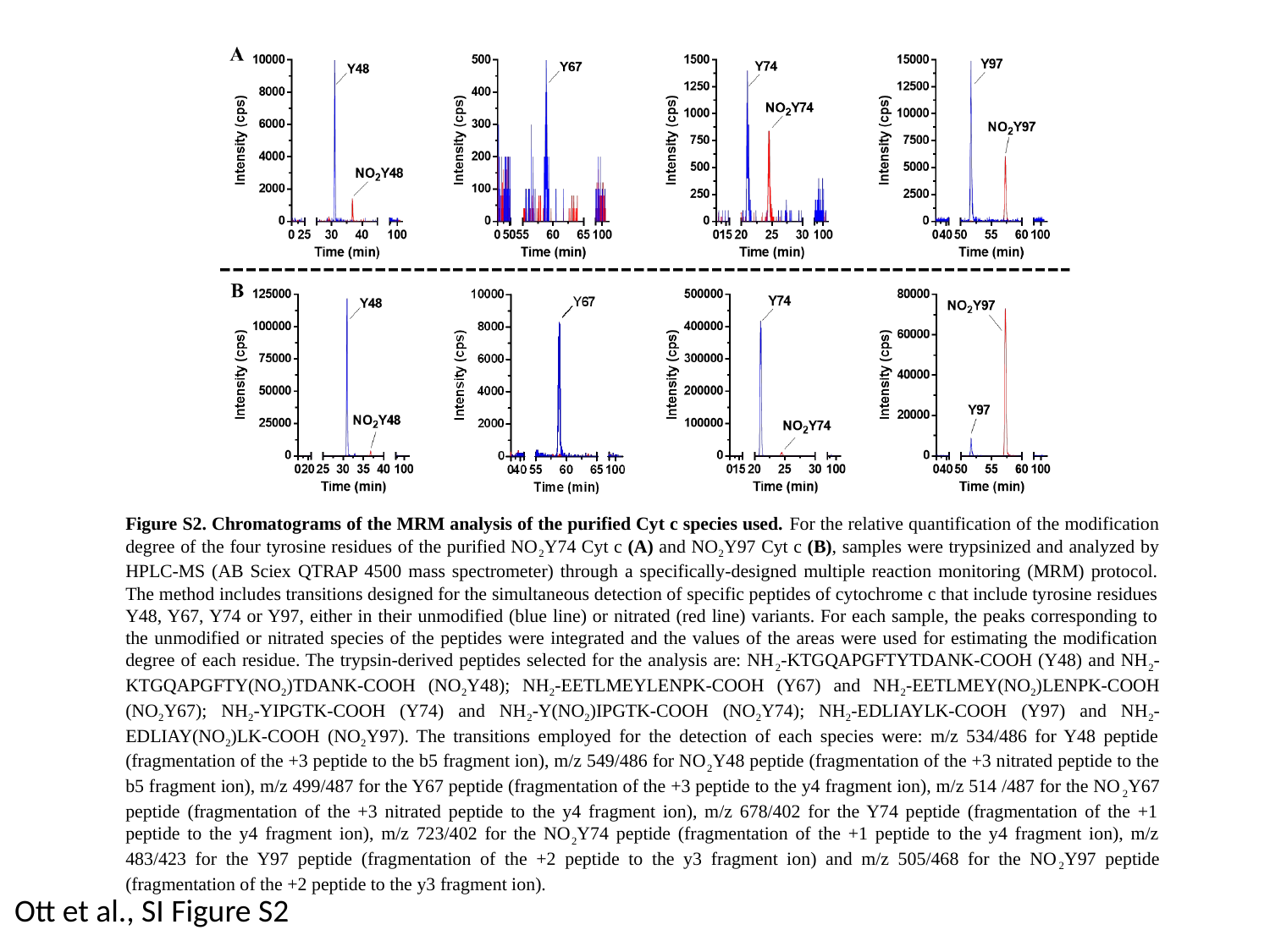

Figure S2. Chromatograms of the MRM analysis of the purified Cyt c species used. For the relative quantification of the modification degree of the four tyrosine residues of the purified NO2Y74 Cyt c (A) and NO2Y97 Cyt c (B), samples were trypsinized and analyzed by HPLC-MS (AB Sciex QTRAP 4500 mass spectrometer) through a specifically-designed multiple reaction monitoring (MRM) protocol. The method includes transitions designed for the simultaneous detection of specific peptides of cytochrome c that include tyrosine residues Y48, Y67, Y74 or Y97, either in their unmodified (blue line) or nitrated (red line) variants. For each sample, the peaks corresponding to the unmodified or nitrated species of the peptides were integrated and the values of the areas were used for estimating the modification degree of each residue. The trypsin-derived peptides selected for the analysis are: NH2-KTGQAPGFTYTDANK-COOH (Y48) and NH2-KTGQAPGFTY(NO2)TDANK-COOH (NO2Y48); NH2-EETLMEYLENPK-COOH (Y67) and NH2-EETLMEY(NO2)LENPK-COOH (NO2Y67); NH2-YIPGTK-COOH (Y74) and NH2-Y(NO2)IPGTK-COOH (NO2Y74); NH2-EDLIAYLK-COOH (Y97) and NH2-EDLIAY(NO2)LK-COOH (NO2Y97). The transitions employed for the detection of each species were: m/z 534/486 for Y48 peptide (fragmentation of the +3 peptide to the b5 fragment ion), m/z 549/486 for NO2Y48 peptide (fragmentation of the +3 nitrated peptide to the b5 fragment ion), m/z 499/487 for the Y67 peptide (fragmentation of the +3 peptide to the y4 fragment ion), m/z 514 /487 for the NO2Y67 peptide (fragmentation of the +3 nitrated peptide to the y4 fragment ion), m/z 678/402 for the Y74 peptide (fragmentation of the +1 peptide to the y4 fragment ion), m/z 723/402 for the NO2Y74 peptide (fragmentation of the +1 peptide to the y4 fragment ion), m/z 483/423 for the Y97 peptide (fragmentation of the +2 peptide to the y3 fragment ion) and m/z 505/468 for the NO2Y97 peptide (fragmentation of the +2 peptide to the y3 fragment ion).
Ott et al., SI Figure S2

## Slide 3
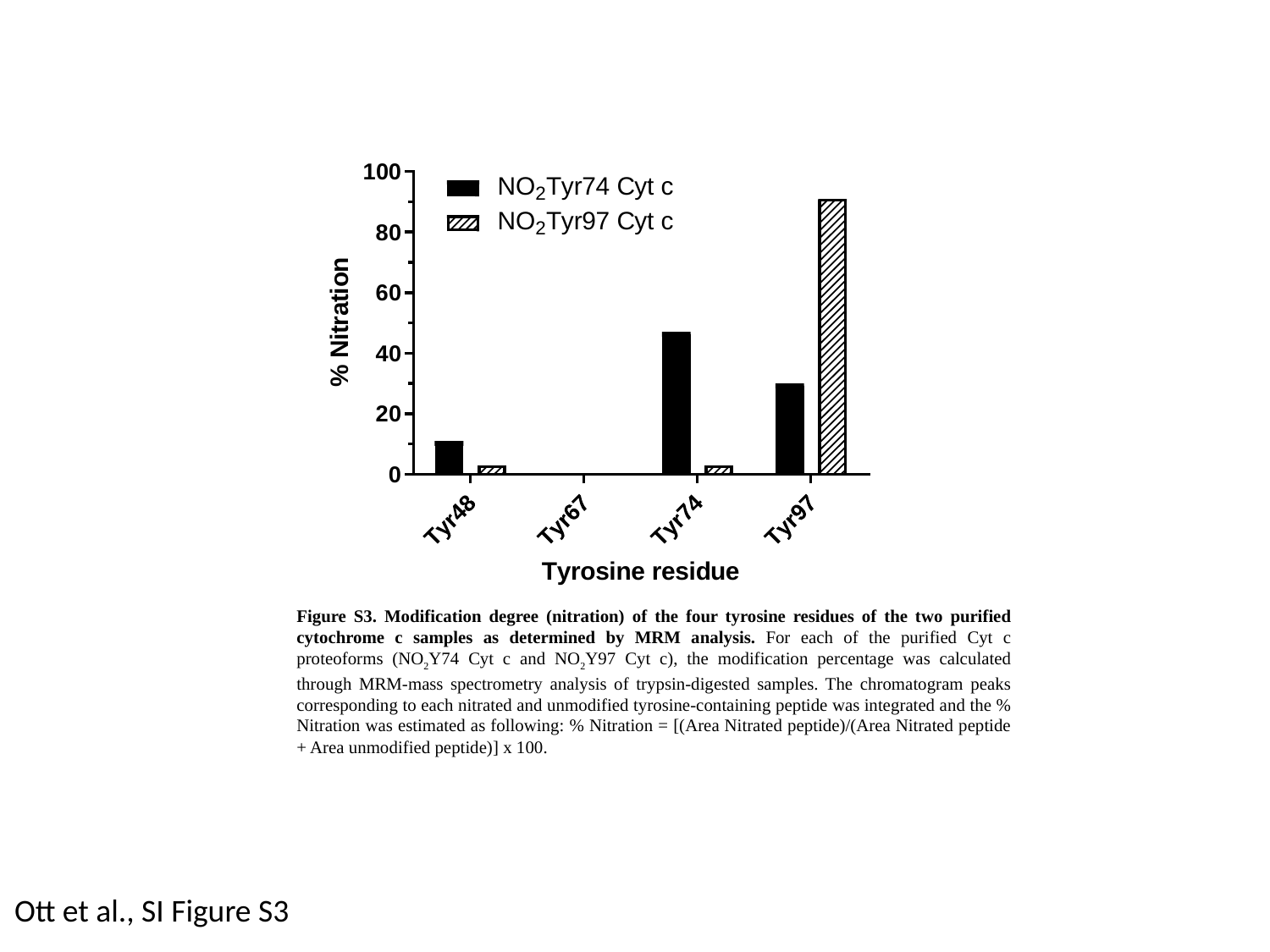

Figure S3. Modification degree (nitration) of the four tyrosine residues of the two purified cytochrome c samples as determined by MRM analysis. For each of the purified Cyt c proteoforms (NO2Y74 Cyt c and NO2Y97 Cyt c), the modification percentage was calculated through MRM-mass spectrometry analysis of trypsin-digested samples. The chromatogram peaks corresponding to each nitrated and unmodified tyrosine-containing peptide was integrated and the % Nitration was estimated as following: % Nitration = [(Area Nitrated peptide)/(Area Nitrated peptide + Area unmodified peptide)] x 100.
Ott et al., SI Figure S3

## Slide 4
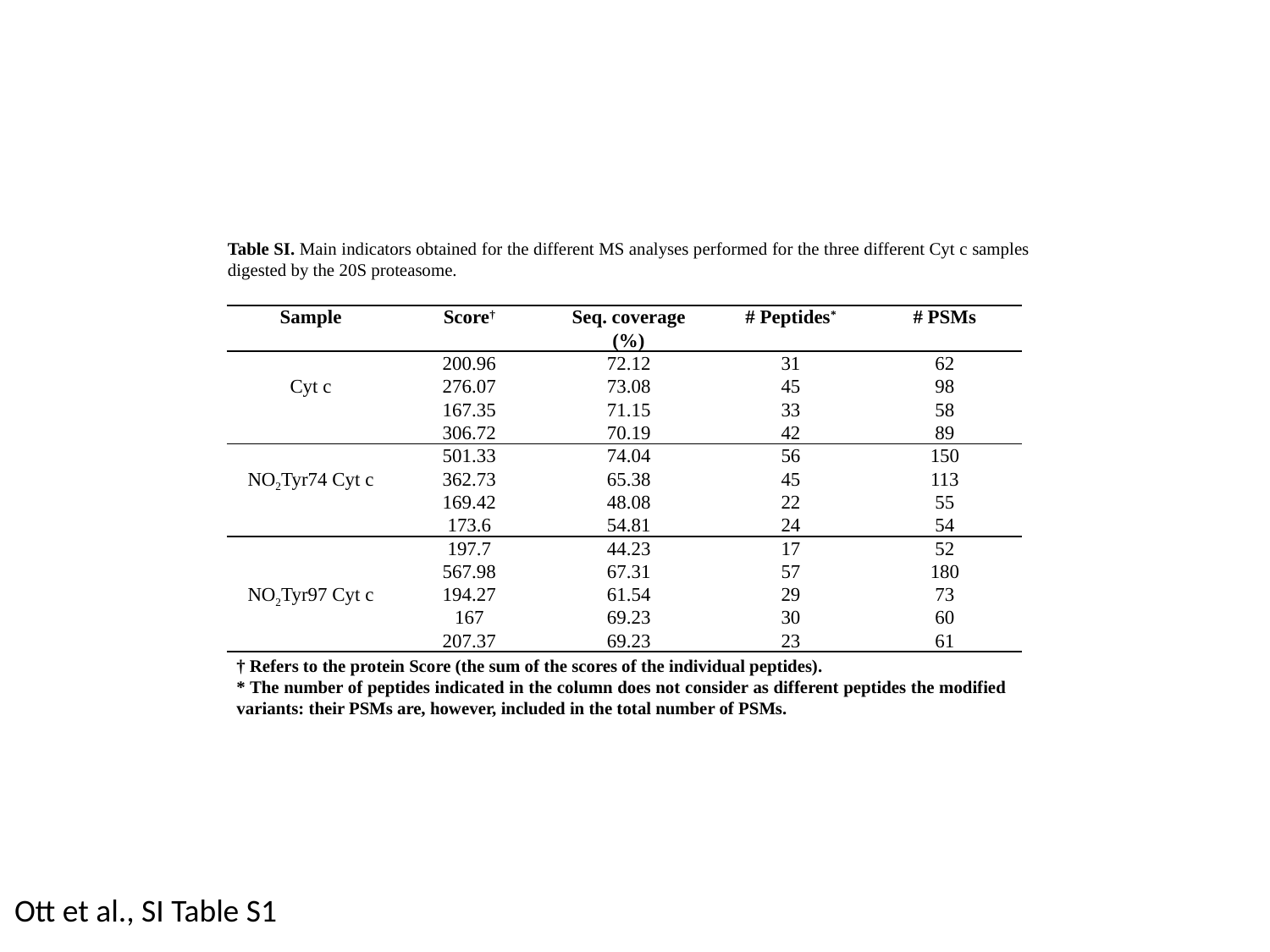

Table SI. Main indicators obtained for the different MS analyses performed for the three different Cyt c samples digested by the 20S proteasome.
| Sample | Score† | Seq. coverage (%) | # Peptides\* | # PSMs |
| --- | --- | --- | --- | --- |
| Cyt c | 200.96 276.07 167.35 306.72 | 72.12 73.08 71.15 70.19 | 31 45 33 42 | 62 98 58 89 |
| NO2Tyr74 Cyt c | 501.33 362.73 169.42 173.6 | 74.04 65.38 48.08 54.81 | 56 45 22 24 | 150 113 55 54 |
| NO2Tyr97 Cyt c | 197.7 567.98 194.27 167 207.37 | 44.23 67.31 61.54 69.23 69.23 | 17 57 29 30 23 | 52 180 73 60 61 |
† Refers to the protein Score (the sum of the scores of the individual peptides).
* The number of peptides indicated in the column does not consider as different peptides the modified variants: their PSMs are, however, included in the total number of PSMs.
Ott et al., SI Table S1

## Slide 5
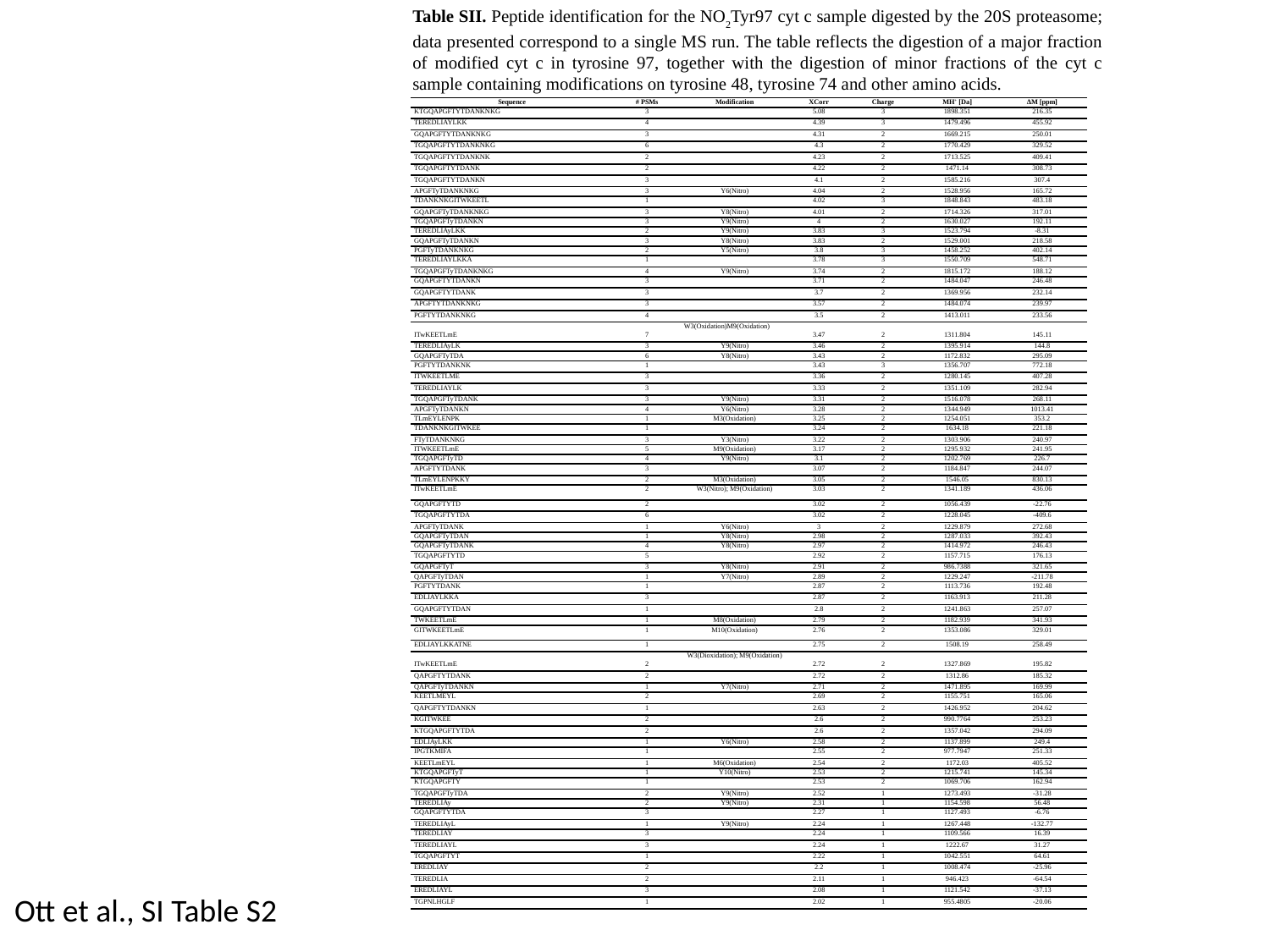

Table SII. Peptide identification for the NO2Tyr97 cyt c sample digested by the 20S proteasome; data presented correspond to a single MS run. The table reflects the digestion of a major fraction of modified cyt c in tyrosine 97, together with the digestion of minor fractions of the cyt c sample containing modifications on tyrosine 48, tyrosine 74 and other amino acids.
| Sequence | # PSMs | Modification | XCorr | Charge | MH+ [Da] | ΔM [ppm] |
| --- | --- | --- | --- | --- | --- | --- |
| KTGQAPGFTYTDANKNKG | 3 | | 5.08 | 3 | 1898.351 | 216.35 |
| TEREDLIAYLKK | 4 | | 4.39 | 3 | 1479.496 | 455.92 |
| GQAPGFTYTDANKNKG | 3 | | 4.31 | 2 | 1669.215 | 250.01 |
| TGQAPGFTYTDANKNKG | 6 | | 4.3 | 2 | 1770.429 | 329.52 |
| TGQAPGFTYTDANKNK | 2 | | 4.23 | 2 | 1713.525 | 409.41 |
| TGQAPGFTYTDANK | 2 | | 4.22 | 2 | 1471.14 | 308.73 |
| TGQAPGFTYTDANKN | 3 | | 4.1 | 2 | 1585.216 | 307.4 |
| APGFTyTDANKNKG | 3 | Y6(Nitro) | 4.04 | 2 | 1528.956 | 165.72 |
| TDANKNKGITWKEETL | 1 | | 4.02 | 3 | 1848.843 | 483.18 |
| GQAPGFTyTDANKNKG | 3 | Y8(Nitro) | 4.01 | 2 | 1714.326 | 317.01 |
| TGQAPGFTyTDANKN | 3 | Y9(Nitro) | 4 | 2 | 1630.027 | 192.11 |
| TEREDLIAyLKK | 2 | Y9(Nitro) | 3.83 | 3 | 1523.794 | -8.31 |
| GQAPGFTyTDANKN | 3 | Y8(Nitro) | 3.83 | 2 | 1529.001 | 218.58 |
| PGFTyTDANKNKG | 2 | Y5(Nitro) | 3.8 | 3 | 1458.252 | 402.14 |
| TEREDLIAYLKKA | 1 | | 3.78 | 3 | 1550.709 | 548.71 |
| TGQAPGFTyTDANKNKG | 4 | Y9(Nitro) | 3.74 | 2 | 1815.172 | 188.12 |
| GQAPGFTYTDANKN | 3 | | 3.71 | 2 | 1484.047 | 246.48 |
| GQAPGFTYTDANK | 3 | | 3.7 | 2 | 1369.956 | 232.14 |
| APGFTYTDANKNKG | 3 | | 3.57 | 2 | 1484.074 | 239.97 |
| PGFTYTDANKNKG | 4 | | 3.5 | 2 | 1413.011 | 233.56 |
| ITwKEETLmE | 7 | W3(Oxidation)M9(Oxidation) | 3.47 | 2 | 1311.804 | 145.11 |
| TEREDLIAyLK | 3 | Y9(Nitro) | 3.46 | 2 | 1395.914 | 144.8 |
| GQAPGFTyTDA | 6 | Y8(Nitro) | 3.43 | 2 | 1172.832 | 295.09 |
| PGFTYTDANKNK | 1 | | 3.43 | 3 | 1356.707 | 772.18 |
| ITWKEETLME | 3 | | 3.36 | 2 | 1280.145 | 407.28 |
| TEREDLIAYLK | 3 | | 3.33 | 2 | 1351.109 | 282.94 |
| TGQAPGFTyTDANK | 3 | Y9(Nitro) | 3.31 | 2 | 1516.078 | 268.11 |
| APGFTyTDANKN | 4 | Y6(Nitro) | 3.28 | 2 | 1344.949 | 1013.41 |
| TLmEYLENPK | 1 | M3(Oxidation) | 3.25 | 2 | 1254.051 | 353.2 |
| TDANKNKGITWKEE | 1 | | 3.24 | 2 | 1634.18 | 221.18 |
| FTyTDANKNKG | 3 | Y3(Nitro) | 3.22 | 2 | 1303.906 | 240.97 |
| ITWKEETLmE | 5 | M9(Oxidation) | 3.17 | 2 | 1295.932 | 241.95 |
| TGQAPGFTyTD | 4 | Y9(Nitro) | 3.1 | 2 | 1202.769 | 226.7 |
| APGFTYTDANK | 3 | | 3.07 | 2 | 1184.847 | 244.07 |
| TLmEYLENPKKY | 2 | M3(Oxidation) | 3.05 | 2 | 1546.05 | 830.13 |
| ITwKEETLmE | 2 | W3(Nitro); M9(Oxidation) | 3.03 | 2 | 1341.189 | 436.06 |
| GQAPGFTYTD | 2 | | 3.02 | 2 | 1056.439 | -22.76 |
| TGQAPGFTYTDA | 6 | | 3.02 | 2 | 1228.045 | -409.6 |
| APGFTyTDANK | 1 | Y6(Nitro) | 3 | 2 | 1229.879 | 272.68 |
| GQAPGFTyTDAN | 1 | Y8(Nitro) | 2.98 | 2 | 1287.033 | 392.43 |
| GQAPGFTyTDANK | 4 | Y8(Nitro) | 2.97 | 2 | 1414.972 | 246.43 |
| TGQAPGFTYTD | 5 | | 2.92 | 2 | 1157.715 | 176.13 |
| GQAPGFTyT | 3 | Y8(Nitro) | 2.91 | 2 | 986.7388 | 321.65 |
| QAPGFTyTDAN | 1 | Y7(Nitro) | 2.89 | 2 | 1229.247 | -211.78 |
| PGFTYTDANK | 1 | | 2.87 | 2 | 1113.736 | 192.48 |
| EDLIAYLKKA | 3 | | 2.87 | 2 | 1163.913 | 211.28 |
| GQAPGFTYTDAN | 1 | | 2.8 | 2 | 1241.863 | 257.07 |
| TWKEETLmE | 1 | M8(Oxidation) | 2.79 | 2 | 1182.939 | 341.93 |
| GITWKEETLmE | 1 | M10(Oxidation) | 2.76 | 2 | 1353.086 | 329.01 |
| EDLIAYLKKATNE | 1 | | 2.75 | 2 | 1508.19 | 258.49 |
| ITwKEETLmE | 2 | W3(Dioxidation); M9(Oxidation) | 2.72 | 2 | 1327.869 | 195.82 |
| QAPGFTYTDANK | 2 | | 2.72 | 2 | 1312.86 | 185.32 |
| QAPGFTyTDANKN | 1 | Y7(Nitro) | 2.71 | 2 | 1471.895 | 169.99 |
| KEETLMEYL | 2 | | 2.69 | 2 | 1155.751 | 165.06 |
| QAPGFTYTDANKN | 1 | | 2.63 | 2 | 1426.952 | 204.62 |
| KGITWKEE | 2 | | 2.6 | 2 | 990.7764 | 253.23 |
| KTGQAPGFTYTDA | 2 | | 2.6 | 2 | 1357.042 | 294.09 |
| EDLIAyLKK | 1 | Y6(Nitro) | 2.58 | 2 | 1137.899 | 249.4 |
| IPGTKMIFA | 1 | | 2.55 | 2 | 977.7947 | 251.33 |
| KEETLmEYL | 1 | M6(Oxidation) | 2.54 | 2 | 1172.03 | 405.52 |
| KTGQAPGFTyT | 1 | Y10(Nitro) | 2.53 | 2 | 1215.741 | 145.34 |
| KTGQAPGFTY | 1 | | 2.53 | 2 | 1069.706 | 162.94 |
| TGQAPGFTyTDA | 2 | Y9(Nitro) | 2.52 | 1 | 1273.493 | -31.28 |
| TEREDLIAy | 2 | Y9(Nitro) | 2.31 | 1 | 1154.598 | 56.48 |
| GQAPGFTYTDA | 3 | | 2.27 | 1 | 1127.493 | -6.76 |
| TEREDLIAyL | 1 | Y9(Nitro) | 2.24 | 1 | 1267.448 | -132.77 |
| TEREDLIAY | 3 | | 2.24 | 1 | 1109.566 | 16.39 |
| TEREDLIAYL | 3 | | 2.24 | 1 | 1222.67 | 31.27 |
| TGQAPGFTYT | 1 | | 2.22 | 1 | 1042.551 | 64.61 |
| EREDLIAY | 2 | | 2.2 | 1 | 1008.474 | -25.96 |
| TEREDLIA | 2 | | 2.11 | 1 | 946.423 | -64.54 |
| EREDLIAYL | 3 | | 2.08 | 1 | 1121.542 | -37.13 |
| TGPNLHGLF | 1 | | 2.02 | 1 | 955.4805 | -20.06 |
Ott et al., SI Table S2
